# Associations between urinary hydration markers and metabolic dysfunction: a cross-sectional analysis of NHANES data, 2008–2010

**DOI:** 10.1007/s00394-021-02575-3

**Published:** 2021-05-18

**Authors:** Tiphaine Vanhaecke, Alberto Dolci, Victor L. Fulgoni, Harris R. Lieberman

**Affiliations:** 1 Health, Hydration and Nutrition Science Department, Danone Research, Route Départementale 128, 91767 Palaiseau, France; 2 Nutrition Impact, LLC, Battle Creek, MI USA; 3 Newton, MA USA

**Keywords:** Diabetes, Metabolic syndrome, Hydration, Urine specific gravity, Urine osmolality, NHANES

## Abstract

**Purpose:**

Growing evidence suggests hydration plays a role in metabolic dysfunction, however data in humans are scarce. This study examined the cross-sectional association between hydration and metabolic dysfunction in a representative sample of the US population.

**Methods:**

Data from 3961 adult NHANES (National Health and Nutrition Examination Survey) participants (49.8% female; age 46.3 ± 0.5 years) were grouped by quartile of urine specific gravity (*U*_SG_, 2007–2008 cohort) or urine osmolality (*U*_Osm_, 2009–2010 cohort) as measures of hydration. Metabolic dysfunction was assessed by glycemic and insulinemic endpoints and by components of the metabolic syndrome. Multivariate-adjusted linear and logistic regression models were used.

**Results:**

Increasing quartiles of *U*_SG_ but not *U*_Osm_ was associated with higher fasting plasma glucose (FPG), glycated hemoglobin (all *P* < 0.01), HOMA-IR and elevated insulin (all *P* < 0.05). Compared with the lowest quartile, those with the highest *U*_SG_ but not *U*_Osm_ had greater risk of metabolic syndrome (Q4 vs. Q1, OR (99% CI): 1.6 (1.0, 2.7), *P* = 0.01) and diabetes (Q4 vs. Q1, OR: 1.8 (1.0, 3.4), *P* < 0.05). Additionally, those with *U*_SG_ > 1.013 or *U*_Osm_ > 500 mOsm/kg, common cut-off values for optimal hydration based on retrospective analyses of existing data, had less favorable metabolic markers. In a subset of participants free from diabetes mellitus, impaired kidney function, hypertension and diuretic medication, *U*_SG_ remained positively associated with FPG (*P* < 0.01) and elevated FPG (*P* < 0.05).

**Conclusion:**

These analyses provide population-based evidence that *U*_SG_ as a proxy for hydration is associated with glucose homeostasis in NHANES 2007–2008. The same association was not significant when *U*_Osm_ was used as a proxy for hydration in the 2009–2010 wave.

**Clinical trial registry:**

Not applicable, as this was a reanalysis of existing NHANES data.

**Supplementary Information:**

The online version contains supplementary material available at 10.1007/s00394-021-02575-3.

## Introduction

High fasting plasma glucose is the key element for the development and diagnosis of diabetes, which is currently among the greatest global public health challenges. The World Health Organization estimates that the rate of increase of individuals with high fasting plasma glucose has surpassed that for obesity, and has designated it as the third highest factor in mortality rate rankings [1]. Metabolic syndrome, a cluster of glucose intolerance, insulin resistance, hypertension and dyslipidemia is also on the rise, thereby magnifying the risk of diabetes incidence [2, 3]. Diet and lifestyle have a direct impact on metabolic function; consequently lifestyle interventions incorporating dietary changes and physical activity may reduce the risk of diabetes in high-risk individuals with impaired glucose regulation [4, 5].

Among potential dietary risk factors, water intake and fluid balance processes have rarely been considered. While it is becoming increasingly evident that increased water intake may decrease the risk of metabolic diseases by reducing intake of sugar-sweetened beverage [6, 7], the hypothesis that water intake or fluid balance processes may play an independent role in modulating disease risk has not received much attention. Several reports have documented an impairment of glucose metabolism by plasma hypertonicity, an indicator of cellular dehydration [8, 9]. In this context, dehydration was suggested to be an additional factor contributing to the development of insulin resistance and risk of diabetes. It is only recently that this hypothesis received further attention, with additional evidence suggesting a link between low water intake and the development of metabolic disease. In the short term, acute low water intake was shown to impair glycemic control in men with type-2 diabetes [10]. In the general population, higher water intake has consistently been associated with lower blood glucose levels and risk of diabetes in men, but not in women [11–13]. In particular, in a prospective cohort on French general population, low water intake was associated with new-onset hyperglycemia over a 9-year follow-up period independently of major baseline confounding factors [11]. The authors also reported water intake was inversely associated with urine specific gravity. When used in large population surveys, urinary hydration biomarkers such as urine osmolality (*U*_Osm_), specific gravity (*U*_SG_), which are non-invasive measures and objective proxy for water homeostasis, may avoid potential recall bias and inaccuracy associated with self-reported recall of food and beverage intake. Both *U*_Osm_ and *U*_SG_ vary according to fluid intake volumes and reflect the end-result of all sources of water intake and water loss, as well as dietary solute load, and represent the diuretic and antidiuretic activity of the kidney [14–17]. However, to-date, urinary biomarkers of hydration have rarely been used to evaluate links between hydration and metabolic health outcomes in large epidemiologic studies.

There is evidence that underhydration is common in the general population; e.g. 70% of the non-acutely ill US population, aged 19–50 years is estimated to have an unmet need for water, as defined by serum sodium outside the normal range as well as a urine osmolality above 500 mOsm/kg [18]. Therefore, the estimate of the risk of metabolic dysfunction in relation to hydration in a representative sample of the general population provides useful information from a public health perspective. Should the hypothesis of a link between hydration and metabolic health be corroborated by causal research, improving hydration by increasing water intake may provide a simple and inexpensive intervention to help prevent the development of metabolic dysfunction.

The first aim of this study was to determine whether there were associations between urinary markers of hydration and metabolic endpoints in a representative sample of the US population, using data from the National Health and Nutrition Examination Survey (NHANES). A second aim of this study was to test whether individuals above common cut-off values for optimal hydration had less favorable metabolic markers. To account for possible reverse causation, analyses were replicated in a cluster of individuals who did not have some conditions likely affecting urinary biomarkers of hydration.

## Methods

### Sample

NHANES is a series of cross-sectional national surveys conducted in the United States by the Centers for Disease Control and Prevention (CDC) and is designed to assess the health and nutritional status of the population using a stratified, nationally representative sampling design. Detailed survey descriptions, methodology, sampling procedures, laboratory test procedures, and data tables are publicly available (www.cdc.gov.nchs/nhanes/). Ethical committee approval for the collection of NHANES data was obtained from the NCHS Research Ethics Review Board in accordance with the Declaration of Helsinki and participants provided written informed consent. Subsequent analyses of de-identified data are permitted by federal regulations on human subjects research and exempt from further IRB review under 45 CFR 46.101(b)(4).

### Data collection and measures

NHANES includes, among other measures, data from a physical examination and a face-to-face structured interview. Sociodemographic variables collected during the interview included age, sex, ethnicity, total income, current smoking status. The poverty income ratio (ratio of household income to the poverty threshold) was used as the indicator of socioeconomic status in the present analysis. Physical activity was categorized in three levels based on self-reported responses of days of vigorous activity (sedentary, 0–3 days per week; moderate 4–6 days per week; and vigorous, 7 days per week). Anthropometric data (including height, weight, and waist circumference) were measured during the physical examination at the mobile examination center (MEC). Urine and blood samples were also collected and processed at the MEC. Sampling and laboratory measurement methods are accessible online (www.cdc.gov.nchs/nhanes/). Urine-specific gravity (2007–2008 cohort) was determined by refractometry (ATAGO PAL-10S, Atago USA, Inc., Bellevue, WA, USA). Urine osmolality (2009–2010 cohort) was measured by freezing point depression osmometer (Osmette II, Precision Systems Inc., Natick, MA, USA). The fasting status of the participants scheduled for morning visits was verified, and laboratory analyses included fasting glucose and insulin, glycated hemoglobin (HbA1c) as well as triglycerides, LDL and HDL cholesterol. Fasting glucose was determined by enzymatic method (hexokinase enzymatic assay). HbA1c was measured by high pressure liquid chromatography (A1c G7 HPLC Glycohemoglobin Analyzer, Tosoh Medics, CA, USA). The analytical method for serum insulin (sandwich ELISA assay, Mercodia, Sweden) was changed part-way through the 2009–2010 cohort (chemiluminescent immunoassay, Elecsys 2010 analyzer, Roche, Switzerland), with a difference in measured insulin values detected between the two methodologies. Using a fractional polynomial regression, the 2010 insulin participant results were increased so they were equivalent to the 2009 insulin results (www.cdc.gov.nchs/nhanes/). HOMA-IR was calculated as glucose*insulin/405 [19]. Triglycerides and HDL-cholesterol were determined by two-reagent enzymatic essay (Modular P chemistry analyzer, Roche, Switzerland). LDL-cholesterol was estimated using the Friedewald equation (Total cholesterol–HDL cholesterol–Triglyceride/5) [20]. Self-reported data on medical conditions and medications were also collected.

### Variable specifications

BMI categories were defined as underweight (BMI < 18.5), normal weight (18.5 ≤ BMI < 25), overweight (25 ≤ BMI < 30) and obese (BMI ≤ 30) [21]. Elevated waist circumference was defined as > 102 cm (male) and > 88 cm (female) [22]; elevated fasting plasma glucose (FPG) ≥ 100 mg/dL (prediabetes) [23] or antidiabetic medication; elevated HbA1c ≥ 6.5% (diabetes) [23]; elevated insulin ≥ 15 µU/L [24] or antidiabetic medication; elevated HOMA-IR ≥ 4.0 [25] or antidiabetic medication; elevated triglycerides ≥ 150 mg/dL [26] or antihyperlipidemic medication; reduced HDL < 40 mg/dL (male) and < 50 mg/dL (female) [27] or antihyperlipidemic medication. Hypertension or elevated blood pressure was defined as systolic ≥ 130 mmHg or diastolic ≥ 80 mmHg [28] or hypertension medication. Participants who reported ever being told that they had diabetes or taking antidiabetic medications were classified as having diabetes. Impaired kidney function was defined as glomerular filtration rate < 60 mL/min/1.73 m^2^ [29]. Participants who had any three of the following criteria were classified as having metabolic syndrome: elevated waist circumference; elevated fasting plasma glucose; elevated triglycerides; reduced HDL; elevated blood pressure) [30].

### Inclusion/exclusion criteria

**Fig. 1:**
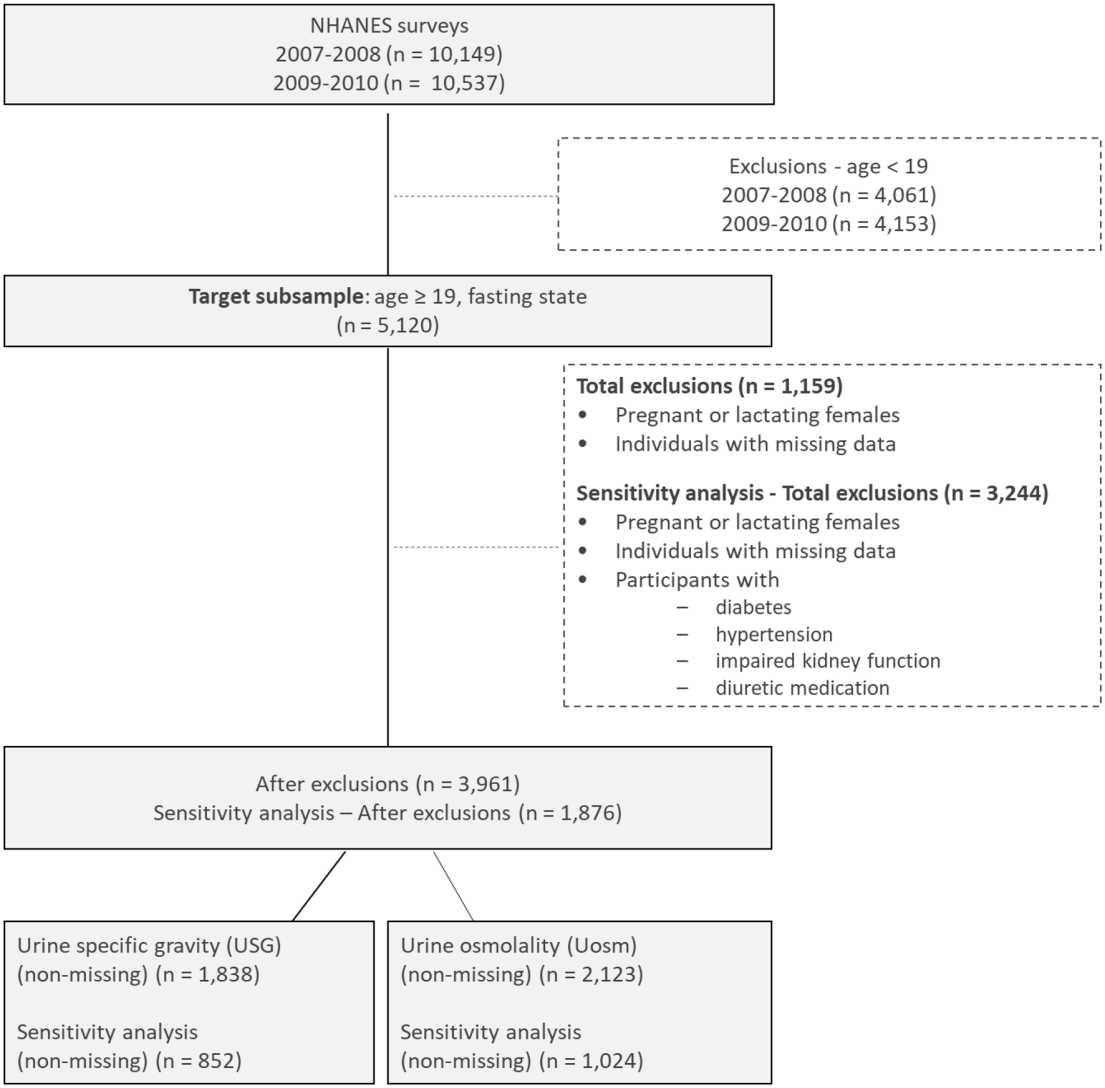
Participant flowchart from the population of adult participants in the NHANES 2007–2008 and 2009–2010

The NHANES 2007–2008 and 2009–2010 fasting subsample data (morning visit at the mobile examination center) of individuals aged ≥ 19 years were used in this study (2007–2008 cohort, *n* = 2424; 2009–2010 cohort *n* = 2696). These were the only NHANES cycles either containing *U*_SG_ or *U*_Osm_ at the time of analyses. After excluding pregnant or lactating females (2007–2008 cohort, *n* = 39; 2009–2010 cohort *n* = 41), as well as individuals missing data for the variables used in this analysis (2007–2008 cohort, *n* = 547; 2009–2010 cohort *n* = 532), the final analysis datasets included *n* = 1838 subjects for the 2007–2008 cohort and *n* = 2123 subjects for the 2009–2010 cohort (Fig. 1). Sample characteristics are shown in Table 1. Additionally, in a sensitivity analysis, individuals from this subsample that had either diabetes, hypertension, impaired kidney function or were taking diuretic medication were further excluded and analyses were repeated on a subgroup of *n* = 852 (2007–2008 cohort) and *n* = 1024 (2009–2010 cohort) otherwise healthy adults.

**Table 1:** Demographic, physiological and anthropometric characteristics of the study population

	2007–2008		2009–2010	
	Mean	SE	Mean	SE
Sample *N*	1838		2123	
Age	45.9	0.7	46.9	0.7
Gender = male (%)	49.8	1.3	50.5	1.0
Gender = female (%)	50.2	1.3	49.5	1.0
Ethnicity = Mexican American (%)	7.9	1.5	8.0	2.0
Ethnicity = Other Hispanic (%)	4.1	0.9	4.3	1.1
Ethnicity = Non-Hispanic White	70.7	3.5	71.3	3.0
Ethnicity = Non-Hispanic Black	11.4	2.3	10.1	1.0
Body mass index (kg/m^2^)	28.3	0.2	28.6	0.2
Underweight (BMI < 18.5) (%)	1.7	0.4	1.6	0.4
Normal weight (18.5 ≤ BMI < 25) (%)	31.0	1.2	29.8	1.8
Overweight (25 ≤ BMI < 30) (%)	34.9	1.5	33.2	1.3
Obese (BMI ≥ 30) (%)	32.5	1.6	35.5	1.6
Smoking = current (%)	20.3	2.2	16.9	1.4
Hypertension (%)	39.4	1.4	40.4	2.2
Glucose (mg/dL)	105.5	0.7	103.2	0.9
Insulin (µU/mL)	11.7	0.4	13.5	0.2
HOMA-IR (glucose*insulin/405)	3.2	0.1	3.6	0.1
HbA1c (%)	5.6	0.03	5.6	0.03
Triglyceride (mg/dL)	123.0	2.2	118.8	2.3
HDL-cholesterol (mg/dL)	53.3	0.7	54.2	0.6
LDL-cholesterol (mg/dL)	115.6	1.1	116.6	1.3
Diabetes (%)	10.4	0.8	10.7	0.7
Metabolic syndrome (%)	41.9	1.5	40.4	1.7
Urine-specific gravity	1.017	0.0002		
Urine osmolality (mOsm/kg)			610	9

Results are sample weighted means or percentages (%) and standard errors (SE) to ensure national representation. Hypertension (systolic ≥ 130 or diastolic ≥ 80 or hypertension medication); diabetes (self-reported diabetes told or taking antidiabetic medications); metabolic syndrome (any three of: elevated waist circumference (> 102 (male), > 88 (female)); elevated fasting plasma glucose (≥ 100 mg/dL or antidiabetic medication); elevated triglycerides (≥ 150 or antihyperlipidemic medication); reduced HDL (< 40 (male), < 50 (female) or antihyperlipidemic medication); elevated blood pressure (systolic ≥ 130 or diastolic ≥ 80 or hypertension medication)

### Statistical analysis

Statistical analyses were performed using SAS 9.4 (Cary, NC, USA). The NHANES fasting subsample survey weights, strata and primary sampling units were used in all analyses using SURVEYMEANS or SURVEYREG procedures of SAS. Respondents were classified by quartiles of *U*_SG_ (2007–2008 cohort) or *U*_Osm_ (2009–2010 cohort), respectively. Because the hydration measurement method for urine concentration was different for the 2007–2008 and 2009–2010 cohorts, results for each cohort are presented separately. Physiologic outcome variables are treated both as continuous variables and presented as adjusted means by quartile of urine concentration, and as incidence measures presented as percentages. We used linear and logistic regression to test whether increasing quartiles of urinary biomarkers of hydration were associated with metabolic endpoints. Regression models included age, sex, ethnicity, poverty income ratio, physical activity level, current smoking status and Body Mass Index (BMI) as covariates to investigate associations independent of these common confounding factors. For example, BMI is a well-described confounder for metabolic variables and is also known to be associated with hydration; people with higher BMI have higher odds of being inadequately hydrated (*U*_Osm_ ≥ 800 mOsm/kg) [31, 32]. Adjusted least square (LS) means, standard errors (SE) and *P* values for quartile trend are presented. Data from logistic regression analyses are expressed as odds ratios (ORs) and 95% confidence intervals (CIs). To test whether individuals above common cut-off values for optimal hydration had less favorable metabolic markers, we explored outcome variables on both sides of a *U*_Osm_ cut-off of 500 mOsm/kg, or a *U*_SG_ of 1.013, since these thresholds have previously been proposed as hydration targets for the general population [33, 34]. Adjusted LS means, SE using regression models with covariates mentioned above are presented and *t*-test were used to assess differences based on cut-offs used.

## Results

### Study population

The present study sample consisted of 1838 individuals in the 2007–2008 cohort and 2123 individuals in the 2009–2010 cohort who had complete information on covariates (Table 1). There were about 50% females both in the 2007–2008 cohort and 2009–2010 cohort. For descriptive purposes, on average, 40–42% of the study population had metabolic syndrome and about 10–11% had self-reported diabetes prior to biological measures at the MEC.

### Associations between *U*_SG_ (2007–2008 cohort) or *U*_Osm_ (2009–2010 cohort) and glycemic endpoints in the general adult population

In the 2007–2008 cohort, fasting plasma glucose (FPG) increased with increasing *U*_SG_ (Table 2, *P* < 0.01), from (mean ± SE) 101.6 ± 0.5 mg/dL in the lowest quartile to 112.1 ± 1.2 mg/dL in the top quartile. HbA1c (%) also increased with increasing *U*_SG_ (*P* < 0.01). This trend was not present in the 2009–2010 cohort, with FPG and HbA1c remaining stable across quartiles of *U*_Osm_ (Table 3).

**Table 2:** Glycemic, insulinemic markers, and metabolic dysfunction endpoints of the adult population of the NHANES 2007–2008 (*n* = 1838) cohort across quartiles of *U*_SG_

Quartiles	Q1	Q2	Q3	Q4	*P* for trend
	*U*_SG_ < 1.011	1.011 ≤ *U*_SG_ < 1.016	1.016 ≤ *U*_SG_ < 1.021	*U*_SG_ ≥ 1.021	
Glycemic markers					
FPG (mg/dL)	101.6 ± 0.5	103.5 ± 0.7	103.9 ± 0.8	112.1 ± 1.2	< 0.01
Elevated FPG	48.9 ± 2.7%	50.8 ± 2.8%	51.8 ± 2.5%	58.0 ± 2.0%	< 0.01
HbA1c (%)	5.50 ± 0.03	5.54 ± 0.03	5.51 ± 0.04	5.67 ± 0.05	< 0.01
Elevated HbA1c	5.5 ± 1.3%	7.4 ± 1.0%	5.8 ± 1.6%	8.9 ± 1.2%	0.09
Insulinemic markers					
Insulin (µU/L)	11.1 ± 0.5	11.7 ± 0.6	11.4 ± 0.5	12.4 ± 0.5	0.12
Elevated insulin	24.1 ± 2.6%	27.6 ± 2.5%	28.0 ± 2.6%	31.7 ± 2.3%	0.04
HOMA-IR	2.9 ± 0.1	3.1 ± 0.2	3.1 ± 0.2	3.6 ± 0.2	0.02
Elevated HOMA-IR	23.1 ± 2.6%	27.0 ± 2.6%	25.5 ± 2.3%	31.1 ± 1.9%	0.04
Other components of the metabolic syndrome					
Elevated waist circumference	53.0 ± 2.3%	48.9 ± 1.7%	53.0 ± 2.2%	52.8 ± 1.4%	0.70
Elevated triglycerides	33.8 ± 2.6%	39.6 ± 1.9%	38.9 ± 2.9%	40.3 ± 1.5%	0.04
Reduced HDL cholesterol	33.1 ± 2.5%	44.2 ± 2.8%	41.4 ± 2.9%	41.9 ± 1.9%	0.02
Elevated BP	43.2 ± 3.0%	38.3 ± 2.2%	38.6 ± 2.6%	37.7 ± 2.3%	0.27

LS means ± standard errors and *P* value for quartile trend are presented. Models were adjusted for age, sex, BMI, ethnicity, poverty income ratio, physical activity level and current smoking status using SURVEYMEANS procedure of SAS. Elevated fasting plasma glucose (FPG) (≥ 100 mg/dL or antidiabetic medication); elevated glycated hemoglobin (HbA1c) (≥ 6.5%); elevated insulin (≥ 15 µU/L or antidiabetic medication); HOMA-IR (glucose*insulin/405); elevated HOMA-IR (≥ 4.0 or antidiabetic medication); elevated waist circumference (> 102 (male), > 88 (female)); elevated triglycerides (≥ 150 or antihyperlipidemic medication); reduced HDL (< 40 (male), < 50 (female) or antihyperlipidemic medication); elevated BP (systolic ≥ 130 or diastolic ≥ 80 or hypertension medication)

**Table 3:** Glycemic, insulinemic markers, and metabolic dysfunction endpoints of the adult population of the NHANES 2009–2010 (*n* = 2123) cohort across quartiles of *U*_Osm_

Quartiles	Q1	Q2	Q3	Q4	*P* for trend
	*U*_Osm_ < 405	405 ≤ *U*_Osm_ < 617	617 ≤ *U*_Osm_ < 808	*U*_Osm_ ≥ 808	
Glycemic markers					
FPG (mg/dL)	102.5 ± 1.1	103.8 ± 1.1	104.3 ± 1.8	102.0 ± 1.1	0.81
Elevated FPG	46.8 ± 2.1%	49.7 ± 2.7%	45.3 ± 2.1%	44.5 ± 3.3%	0.37
HbA1c (%)	5.60 ± 0.04	5.62 ± 0.02	5.63 ± 0.06	5.55 ± 0.03	0.27
Elevated HbA1c	6.7 ± 1.0%	9.4 ± 1.2%	7.4 ± 1.9%	6.0 ± 1.0%	0.32
Insulinemic markers					
Insulin (µU/L)	13.2 ± 0.2	13.3 ± 0.6	13.5 ± 0.7	13.8 ± 0.4	0.35
Elevated insulin	34.3 ± 2.3%	33.7 ± 3.6%	33.9 ± 2.4%	35.2 ± 2.0%	0.79
HOMA-IR	3.5 ± 0.1	3.6 ± 0.2	3.6 ± 0.2	3.6 ± 0.1	0.62
Elevated HOMA-IR	33.4 ± 1.9%	32.9 ± 3.2%	30.8 ± 2.4%	33.0 ± 1.8%	0.71
Other components of the metabolic syndrome					
Elevated waist circumference	53.3 ± 2.1%	53.7 ± 1.9%	54.5 ± 1.7%	55.1 ± 2.0%	0.43
Elevated triglycerides	35.1 ± 1.8%	36.8 ± 2.2%	35.6 ± 2.3%	34.9 ± 4.2%	0.92
Reduced HDL cholesterol	43.0 ± 2.3%	46.2 ± 2.2%	38.9 ± 2.1%	39.7 ± 3.8%	0.20
Elevated BP	44.7 ± 2.1%	42.0 ± 2.4%	40.8 ± 3.0%	34.4 ± 2.7%	< 0.01

LS means ± standard errors and *P* value for quartile trend are presented. Models were adjusted for age, sex, BMI, ethnicity, poverty income ratio, physical activity level and current smoking status using SURVEYMEANS procedure of SAS. Elevated fasting plasma glucose (FPG) (≥ 100 mg/dL or antidiabetic medication); elevated glycated hemoglobin (HbA1c) (≥ 6.5%); elevated insulin (≥ 15 µU/L or antidiabetic medication); HOMA-IR (glucose*insulin/405); elevated waist circumference (> 102 (male), > 88 (female)); elevated HOMA-IR (≥ 4.0 or antidiabetic medication); elevated triglycerides (≥ 150 or antihyperlipidemic medication); reduced HDL (< 40 (male), < 50 (female) or antihyperlipidemic medication); elevated BP (systolic ≥ 130 or diastolic ≥ 80 or hypertension medication)

Higher *U*_SG_ (2007–2008 cohort) but not higher *U*_Osm_ (2009–2010 cohort) was also associated with reaching glycemic diagnostic criteria for impaired fasting glucose (Table 2, *P* < 0.01). Individuals in the highest vs. the lowest quartiles for *U*_SG_ were more likely to reach thresholds for elevated FPG (58.0 ± 2.1% vs. 48.9 ± 2.7%, *P* < 0.01).

### Associations between *U*_SG_ or *U*_Osm_ and insulinemic endpoints

In the 2007–2008 cohort, insulin resistance (HOMA-IR), increased with urine concentration (Table 2, *P* < 0.05). No statistical association was found between *U*_Osm_ and insulinemic endpoints (Table 3).

### Associations between *U*_SG_ or *U*_Osm_ and components of the metabolic syndrome

In addition to a higher prevalence of elevated FPG, reduced HDL cholesterol was associated with increasing *U*_SG_ (Table 2, *P* < 0.05). Elevated blood pressure was negatively associated with *U*_Osm_ (Table 2, *P* < 0.01) but not with *U*_SG_ (Table 2)_._ Finally, elevated triglycerides and waist circumference were not associated with urine concentration assessed either by *U*_SG_ or *U*_Osm_ (*U*_SG_: Table 2, *U*_Osm_: Table 3).

### Associations of *U*_SG_ and *U*_Osm_ with glycemic and insulinemic endpoints and metabolic syndrome in the adult population deemed healthy

In the segment of the population free from diabetes mellitus, impaired kidney function, hypertension and diuretic medication, conditions which are known to affect hydration status, *U*_SG_ but not *U*_Osm_ was positively associated with FPG and elevated FPG (*P* < 0.01 and *P* < 0.05, respectively) (*U*_SG_: Table 4; *U*_Osm_: Table 5).

**Table 4:** Glycemic, insulinemic markers, and metabolic dysfunction endpoints of the adult population of the NHANES 2007–2008 cohort free from diabetes mellitus, impaired kidney function, hypertension and diuretic medication (*n* = 852) across quartiles of *U*_SG_

Quartiles	Q1	Q2	Q3	Q4	*P* for trend
	*U*_SG_ < 1.011	1.011 ≤ *U*_SG_ < 1.016	1.016 ≤ *U*_SG_ < 1.021	*U*_SG_ ≥ 1.021	
Glycemic markers					
FPG (mg/dL)	96.3 ± 0.6	96.8 ± 0.8	97.1 ± 0.8	99.0 ± 0.6	< 0.01
Elevated FPG	33.5 ± 4.1%	32.9 ± 3.5%	37.0 ± 4.0%	42.2 ± 2.9%	0.03
HbA1c (%)	5.32 ± 0.02	5.29 ± 0.03	5.29 ± 0.03	5.26 ± 0.03	0.13
Elevated HbA1c	0.8 ± 0.6%	0.1 ± 0.1%	0.1 ± 0.1%	0.8 ± 0.4%	0.97
Insulinemic markers					
Insulin (µU/L)	10.0 ± 0.5	9.5 ± 0.6	10.1 ± 0.4	10.6 ± 0.6	0.32
Elevated insulin	16.4 ± 3.1%	13.2 ± 2.6%	19.8 ± 2.9%	17.3 ± 3.1%	0.53
HOMA-IR	2.4 ± 0.1	2.3 ± 0.2	2.5 ± 0.1	2.7 ± 0.2	0.24
Elevated HOMA-IR	14.5 ± 2.9%	12.5 ± 2.7%	17.5 ± 2.0%	16.4 ± 3.0%	0.46
Other components of the metabolic syndrome					
Elevated waist circumference	39.4 ± 3.1%	35.7 ± 2.6%	41.8 ± 4.0%	42.0 ± 1.9%	0.27
Elevated triglycerides	20.5 ± 3.7%	20.8 ± 2.9%	22.2 ± 2.8%	27.8 ± 2.9%	0.14
Reduced HDL cholesterol	25.4 ± 3.9%	34.9 ± 4.6%	32.9 ± 4.2%	33.2 ± 3.9%	0.19

LS means ± standard errors and *P* value for quartile trend are presented. Models were adjusted for age, sex, BMI, ethnicity, poverty income ratio, physical activity level and current smoking status using SURVEYMEANS procedure of SAS. Elevated fasting plasma glucose (FPG) (≥ 100 mg/dL or antidiabetic medication); elevated glycated hemoglobin (HbA1c) (≥ 6.5%); elevated insulin (≥ 15 µU/L or antidiabetic medication); HOMA-IR (glucose*insulin/405); elevated waist circumference (> 102 (male), > 88 (female)); elevated HOMA-IR (≥ 4.0 or antidiabetic medication); elevated triglycerides (≥ 150 or antihyperlipidemic medication); reduced HDL (< 40 (male), < 50 (female) or antihyperlipidemic medication)

**Table 5:** Glycemic, insulinemic markers, and metabolic dysfunction endpoints of the adult population of the NHANES 2009–2010 cohort free from diabetes mellitus, impaired kidney function, hypertension and diuretic medication (*n* = 1024) across quartiles of *U*_Osm_

Quartiles	Q1	Q2	Q3	Q4	*P* for trend
	*U*_Osm_ < 376	376 ≤ *U*_Osm_ < 653	653 ≤ *U*_Osm_ < 852	*U*_Osm_ ≥ 852	
Glycemic markers					
FPG (mg/dL)	97.0 ± 0.8	96.4 ± 1.0	95.6 ± 0.9	95.2 ± 0.7	0.06
Elevated FPG	32.1 ± 2.7%	35.4 ± 3.2%	28.1 ± 3.1%	28.5 ± 3.8%	0.28
HbA1c (%)	5.41 ± 0.02	5.32 ± 0.03	5.35 ± 0.02	5.30 ± 0.03	0.04
Elevated HbA1c	0.7 ± 0.3%	1.0 ± 0.7%	0.9 ± 0.8%	0.2 ± 0.3%	0.37
Insulinemic markers					
Insulin (µU/L)	11.7 ± 0.7	10.8 ± 0.5	11.5 ± 0.5	12.0 ± 0.7	0.64
Elevated insulin	20.6 ± 2.0%	16.8 ± 2.4%	23.7 ± 2.6%	24.6 ± 3.4%	0.20
HOMA-IR	2.9 ± 0.2	2.6 ± 0.1	2.8 ± 0.2	2.9 ± 0.2	0.78
Elevated HOMA-IR	20.6 ± 1.8%	15.3 ± 2.6%	16.2 ± 2.5%	20.7 ± 3.2%	0.95
Other components of the metabolic syndrome					
Elevated waist circumference	37.3 ± 1.4%	43.1 ± 3.4%	41.2 ± 2.6%	39.6 ± 1.6%	0.57
Elevated triglycerides	19.4 ± 2.7%	24.2 ± 3.6%	29.4 ± 2.9%	22.1 ± 4.5%	0.14
Reduced HDL cholesterol	33.6 ± 3.1%	36.7 ± 5.1%	29.4 ± 2.9%	25.3 ± 3.6%	0.09

LS means ± standard errors and *P* value for quartile trend are presented. Models were adjusted for age, sex, BMI, ethnicity, poverty income ratio, physical activity level and current smoking status using SURVEYMEANS procedure of SAS. Elevated fasting plasma glucose (FPG) (≥ 100 mg/dL or antidiabetic medication); elevated glycated hemoglobin (HbA1c) (≥ 6.5%); elevated insulin (≥ 15 µU/L or antidiabetic medication); HOMA-IR (glucose*insulin/405); elevated waist circumference (> 102 (male), > 88 (female)); elevated HOMA-IR (≥ 4.0 or antidiabetic medication); elevated triglycerides (≥ 150 or antihyperlipidemic medication); reduced HDL (< 40 (male), < 50 (female) or antihyperlipidemic medication)

### Odds ratios for metabolic disease

**Fig. 2:**
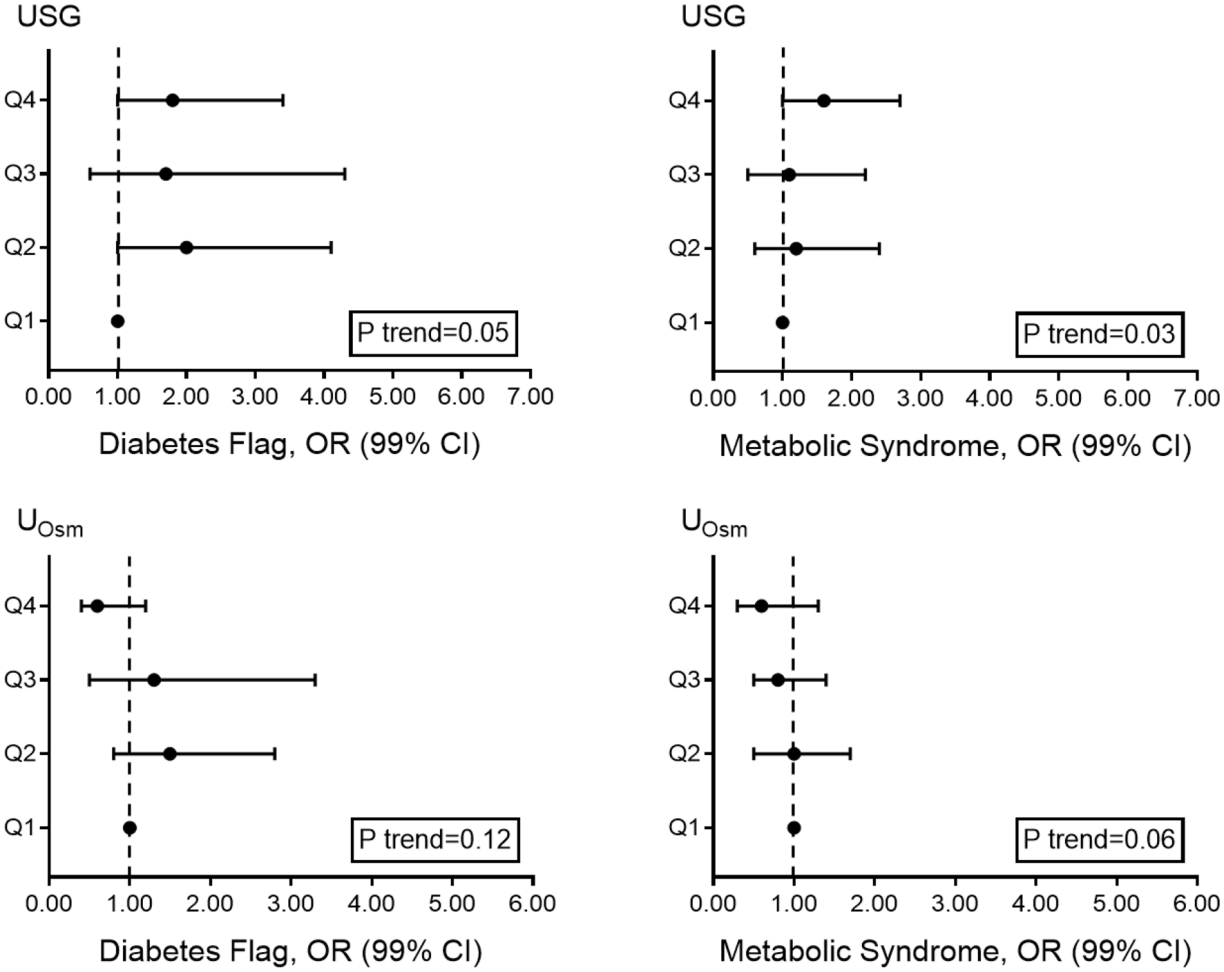
Adjusted odds ratios (OR) and 99% confidence interval (CI) for metabolic syndrome and diabetes mellitus in relation to quartiles of *U*_SG_ (*N* = 1838) and *U*_Osm_ (*N* = 2123). OR were adjusted for age, sex, BMI, ethnicity, poverty income ratio, physical activity level and current smoking status. Diabetes flag (diabetes told or antidiabetic medication); metabolic syndrome (any three of: elevated waist circumference (> 102 (male), > 88 (female)); elevated fasting plasma glucose (FPG) (≥ 100 mg/dL or antidiabetic medication); elevated triglycerides (≥ 150 or antihyperlipidemic medication); reduced HDL (< 40 (male), < 50 (female) or antihyperlipidemic medication); elevated blood pressure (BP) (systolic ≥ 130 or diastolic ≥ 80 or hypertension medication)); odds ratios (OR); Quartile (Q)

Higher *U*_SG_ was associated with increased odds of diabetes (Q4 vs. Q1, OR (99% CI): 1.8 (1.0, 3.4), *P* = 0.02) and metabolic syndrome (Q4 vs. Q1, OR: 1.6 (1.0, 2.7), *P* = 0.01) (Fig. 2, Table S1 (URL: https://figshare.com/s/9521e8960e6e30550bdc; https://doi.org/10.6084/m9.figshare.12783413) in the NHANES 2007–2008 cohort. In the segment of the population free from diabetes mellitus, impaired kidney function, hypertension and diuretic medication, higher *U*_SG_ remained associated with increased odds of metabolic syndrome (Q4 vs. Q1, OR: 2.7 (1.1, 6.6), *P* < 0.01) (Table S1). There was no association between *U*_Osm_ and odds of diabetes or metabolic syndrome in the NHANES 2009–2010 cohort.

### Differences in metabolic markers between well-hydrated (*U*_Osm_ ≤ 500 mOsm/kg; *U*_SG_ ≤ 1.013) and under-hydrated (*U*_Osm_ > 500 mOsm/kg; *U*_SG_ > 1.013) individuals

Compared to those with *U*_SG_ ≤ 1.013, individuals with higher *U*_SG_ had higher glucose (Table 6, *P* < 0.01) whereas individuals with *U*_Osm_ > 500 mOsm/kg had higher insulin (*P* < 0.01) compared to those with lower *U*_Osm_. After excluding participants with diabetes mellitus, impaired kidney function, hypertension and diuretic medication, *U*_SG_ remained positively associated with glucose (*P* < 0.01).

**Table 6:** Glycemic and insulinemic markers in relation to *U*_SG_ and *U*_Osm_ thresholds for optimal hydration

	*U*_SG_			*U*_Osm_		
	≤ 1.013	> 1.013	*P* value	≤ 500 mOsm/kg	> 500 mOsm/kg	*P* value
*Glycemic markers*						
FPG (mg/dL)						
Study population	101.8 ± 0.6	107.4 ± 0.6	< 0.01	101.9 ± 1.0	103.8 ± 1.0	0.05
Healthy subsample	95.9 ± 0.5	98.1 ± 0.5	< 0.01	96.6 ± 0.6	95.8 ± 0.7	0.27
HbA1c (%)						
Study population	5.51 ± 0.02	5.58 ± 0.03	0.06	5.58 ± 0.04	5.61 ± 0.02	0.48
Healthy subsample	5.3 ± 0.02	5.3 ± 0.02	0.43	5.4 ± 0.02	5.3 ± 0.01	0.08
*Insulinemic markers*						
Insulin (µU/L)						
Study population	11.2 ± 0.4	12.0 ± 0.4	0.12	13.3 ± 0.4	14.2 ± 0.3	< 0.01
Healthy subsample	9.7 ± 0.4	10.2 ± 0.4	0.40	11.5 ± 0.5	11.5 ± 0.4	0.96
HOMA-IR						
Study population	2.9 ± 0.1	3.3 ± 0.1	0.02	3.5 ± 0.2	3.6 ± 0.1	0.78
Healthy subsample	2.4 ± 0.1	2.5 ± 0.1	0.30	2.8 ± 0.2	2.8 ± 0.1	0.93

LS means ± standard errors and *P* values from *t*-tests are presented. Models are adjusted for age, sex, BMI, ethnicity, poverty income ratio, physical activity level and current smoker status in the study population (*U*_SG_: 2007–2008 (*n* = 1838); *U*_Osm_: 2009–2010 (*n* = 2123)) and in a subset of participants free from diabetes mellitus, impaired kidney function, hypertension and diuretic medication (*U*_SG_: 2007–2008 (*n* = 852); *U*_Osm_: 2009–2010 (*n* = 1024)); fasting plasma glucose (FPG); glycated hemoglobin (HbA1c); HOMA-IR (glucose*insulin/405)

## Discussion

This cross-sectional analysis of a representative sample of the US population revealed significant relationships between hydration and some measures of metabolic health. While several previous investigations have examined relationships between water, fluid intake [10, 11, 13] or vasopressin (copeptin), a key hormone in the regulation of body fluids [35–37] and metabolic outcomes, urinary biomarkers of hydration have seldom been studied in relation to metabolic outcomes [18, 31, 38].

Specifically, we show that in both the general population as well as in a subset of healthy individuals, more concentrated urine (as measured by specific gravity) was associated with some glycemic and insulinemic markers. Puzzlingly, these associations were not replicated in the subsequent NHANES cohort, where only a few relationships between hydration and metabolic outcomes were found to be significant. These latter results were consistent with a recent analysis of NHANES 2009–2012 which showed that the prevalence of underhydration, defined as serum sodium > 145 mmol/L, spot urine volume < 50 mL, and/or spot urine osmolality ≥ 500 mmol/kg, was not higher among individuals with diabetes, elevated glucose or elevated HbA1c than among individuals without these conditions [18]. Although *U*_SG_ and *U*_Osm_ are well correlated under normal physiological conditions [39], the inherent differences that exist between urine osmolality and urine specific gravity may partly explain the discrepancies found between the two cohorts. Urine osmolality is the number of molecules per kilogram of water contained in urine while urine specific gravity is a comparison of the density of urine to that of water [40]. Therefore, specific gravity is affected by the number of molecules and their molecular weights while osmolality is not. In most cases, *U*_Osm_ and *U*_SG_ are linearly correlated but if many high-molecular-weight molecules are present in the urine, *U*_SG_ will overestimate the urine solute concentration, whereas *U*_Osm_ remains accurate [40]. Additionally, because *U*_SG_ and *U*_Osm_ present a different distribution, where the former is linear, and the latter is exponential we could expect that where there are associations with one, there are not necessarily associations with the other. Finally, by accounting for confounding factors and having provided cut-off values, we excluded the values at the 2-ends of the distribution where *U*_Osm_ shows most of the differences compared to *U*_SG_.

Overall, these findings provide additional population-based evidence on physiological pathways which link water intake, changes in circulating vasopressin (AVP) and metabolic health [41]. It is well established that the end-result of antidiuretic activity of AVP acting on the kidney is maintenance of water balance in response to varying levels of water intake and loss and is well reflected by urine concentration, measured by osmolality, specific gravity, or color [14–17, 42, 43]. For example, the concentration of AVP has been shown to differ between low- and high-drinkers [16, 44], and it has recently been reported that increasing water intake can reduce plasma AVP/copeptin, both in the short term (over several hours or days) [44, 45], and over several weeks [46].

The evidence that concentrated urine is associated with a worse glycemic profile is consistent with recent findings from another large population-based sample, the Malmö Offspring Study, in which high urine osmolality was associated with unfavorable glucometabolic profile [38]. Additionally, an observational study which primarily aimed to describe the determinants of urine osmolality (medical condition, socio-demographic and lifestyle factors) using the NHANES 2009–2012 cohort reported lower blood glucose in participants with very diluted urine but did not explore this association in multivariate adjusted models [31]. However, the study reported no association between the multivariate adjusted odds ratios for very dilute or very concentrated urine and diabetes, a finding in line with our results. Additionally, elevated blood glucose (hyperglycemia, elevated HbA1c) was recently associated with higher copeptin in recent a cross-sectional study [38], and the reduction in blood glucose following water supplementation was found to be driven by individuals with higher baseline copeptin and greater copeptin reduction in a recent small intervention study [47]. However, while many studies have observed links between metabolic health and evidence of underhydration or challenges to water homeostasis, whether it is measured by water intake, urine concentration or copeptin (AVP), the evidence remains inconsistent. Evidence of associations between water intake and glycemic parameters vary depending on the glycemic status of the population studied, the severity of the glucometabolic disorder and sex. In normoglycemic men or in men free from diabetes, water intake is inversely and independently associated with the risk of developing hyperglycemia [11] and associated with lower likelihood of having elevated HbA1c [13]; whereas in studies that did not exclude subjects based on glycemic parameters, no associations were found between glycemic parameters and water intake [11, 38]. Collectively, these findings suggest a potential relationship between water intake and glucometabolic disorders, with a stronger association in men than women.

A body of research also has found positive associations between euhydration or water homeostasis and favorable insulinemic profile [10, 37, 48, 49]. To our knowledge, our study was the first population-based study to specifically explore urine concentration in relation to metabolic endpoints in multivariate adjusted models. In addition to measures of insulinemic and glycemic parameters, evidence of underhydration and challenges to water homeostasis have been associated with several components of metabolic syndrome, including adiposity (higher waist circumference or abdominal obesity) [38, 49–51] and dyslipidemia (lower HDL cholesterol, higher triglycerides) [38, 49, 51] in multivariate adjusted models. Our current study also found positive associations between a marker of adiposity (lower HDL cholesterol) or the risk of metabolic syndrome and *U*_SG_.

Finally, it should also be noted that in individuals with higher copeptin, the risk of developing diabetes may be increased, even after adjusting for a wide range of confounding factors [36, 48–50, 52]. This is observed even in subsets of normoglycemic individuals at baseline [36, 48, 52]. Abbasi et al. reported sex differences in the PREVEND cohort, with the association between plasma copeptin and incident diabetes found in women but not in men [52]. On the contrary, Pan et al. [12] reported no decreased risk of type-2 diabetes with increased water consumption in a large, female-only cohort. While these two findings provide supportive evidence, they are difficult to compare as the former used plasma copeptin and the latter used self-reported water intake as independent variables. This inconsistency suggests that additional studies should be conducted to clarify how water and hydration influence diabetes risk in men and women.

There are areas in which the existing findings are conflicting or inconclusive. Many of the associations between *U*_SG_ and metabolic outcomes in the 2007–2008 cohort were not found in the next cohort; for example, fasting plasma glucose and the incidence of diabetes were related to *U*_SG_ in 2007–2008, but this finding was not confirmed in the 2009–2010 cohort which used *U*_Osm_ as a measure of urine concentration. This explains why results for each cohort are presented separately and is perhaps one of the factors contributing to the lack of consistency in our findings. Furthermore, the analytical method for serum insulin was changed part-way through the 2009–2010 cohort, with a difference in measured insulin values detected between the two methodologies (www.cdc.gov.nchs/nhanes/). While a correction factor was applied to the values obtained in 2010 to bring them in line with the 2009 values, this may be another source of imprecision that may have contributed to the inconsistency in results. We also acknowledge the limitations of a single, morning, spot urine sample as an objective proxy for hydration: morning urine samples are less likely to represent 24-h urine concentration, which is more reflective of fluid intake. This may further explain the discrepancies in the associations observed between hydration and metabolic outcomes between the two cohorts. However, because of the nature of the metabolic outcomes we were interested in studying, only participants having completed fasted-state, morning visits (and thus providing morning urine samples) were included in this analysis. To minimize the impact of this limitation, analyses by quartile of urine concentration were conducted, as it has been shown that low-volume drinkers have significantly higher urine concentration than high-volume drinkers, even in morning samples [16, 17]. Moreover, spot urine samples are the most practical measure of hydration in such large cohorts and provide an opportunity to study a relevant hydration biomarker linked to fluid intake, AVP, and metabolic health outcomes in large representative population samples. Although our analyses have consistently adjusted for a wide range of demographic, socio-economic, lifestyle and biological confounding factors, relevant environmental factors such as the season of the examination, and dietary factors such as salt intake, protein intake or total energy intake were not considered in this analysis and may have affected the results. There were also issues of residual confounders due to events that occurred prior to conduct of the current analysis. Current smoking status was considered, thereby ignoring any change in smoking behavior in previous years. As for any cross-sectional study, there is a risk of reverse causation. However, exclusion of individuals with either diabetes, hypertension, impaired kidney function or taking diuretic medication in a sensitivity analysis is likely to have limited the risk of reverse causation. Finally, one of the strengths of this study is that it is an analysis of a nationally representative sample of the population and uses objective urinary biomarkers of hydration that provide an accurate measure of hydration at the population level.

Future research should establish whether a causal link exists between high urine concentration due to low or insufficient fluid intake and metabolic dysfunction. Furthermore, studies should be conducted to determine whether these physiological indicators can be used to define a target fluid intake for optimal hydration that compensates for water losses and maintains urine output that reduces the risk of metabolic dysfunction.

## Supplementary Information

Below is the link to the electronic supplementary material.

Supplementary file1 (PDF 143 KB)

## Data Availability

Detailed NHANES survey descriptions, methodology, sampling procedures, laboratory test procedures, and data tables are publicly available (www.cdc.gov.nchs/nhanes/).

## Abbreviations

AVP: Arginine vasopressin
CDC: Centers for Disease Control and Prevention
FPG: Fasting plasma glucose
HbA1c: Glycated haemoglobin
LS: Least square
NHANES: National Health and Nutrition Examination Survey
NCHS: National Center for Health Statistics
*U*_Osm_: Urine osmolality
*U*_SG_: Urine specific gravity
V1aR: Vasopressin 1A receptor
WHO: World Health Organisation
