# Supplementary material for: Associations between urinary hydration markers and metabolic dysfunction: a cross-sectional analysis of NHANES data, 2008–2010
Source: Eur J Nutr. 2021 May 18;60(8):4229–41. doi: 10.1007/s00394-021-02575-3 (PMC8572195; doi:10.1007/s00394-021-02575-3)
Supplement: Supplementary file 1 — Supplementary file1 (PDF 143 KB) [file 394_2021_2575_MOESM1_ESM.pdf]

Table S1. Adjusted odds ratios (OR) and 99% lower- and upper confidence limit (LCL-UCL) for diabetes mellitus and metabolic syndrome in relation to quartiles (Q) of U_{SG} and U_{Osm}

	Population	n	Q2 U_{SG} OR (LCL-UCL)	P*	Q3 U_{SG} OR (LCL-UCL)	P*	Q4 U_{SG} OR (LCL-UCL)	P*	Quartile trend P
Diabetes flag	Study population	1,838	2.0 (1.0–4.1)	0.01	1.7 (0.6–4.3)	0.14	1.8 (1.0–3.4)	0.02	0.05
Metabolic syndrome	Study population	1,838	1.2 (0.6–2.4)	0.50	1.1 (0.5–2.2)	0.85	1.6 (1.0–2.7)	0.01	0.03
	Healthy subsample	852	1.5 (0.7–3.3)	0.19	1.2 (0.4–3.5)	0.57	2.7 (1.1–6.6)	< 0.01	0.02

	Population	n	Q2 U_{Osm} OR (LCL-UCL)	P*	Q3 U_{Osm} OR (LCL-UCL)	P*	Q4 U_{Osm} OR (LCL-UCL)	P*	Quartile trend P
Diabetes flag	Study population	2,123	1.5 (0.8–2.8)	0.12	1.3 (0.5–3.3)	0.37	0.6 (0.4–1.2)	0.04	0.12
Metabolic syndrome	Study population	2,123	1.0 (0.5–1.7)	0.82	0.8 (0.5–1.4)	0.28	0.6 (0.3–1.3)	0.08	0.06
	Healthy subsample	1,024	1.0 (0.4–2.4)	0.95	1.0 (0.5–2.0)	0.93	0.8 (0.2–2.8)	0.56	0.57

OR adjusted for adjusted for age, sex, BMI, ethnicity, poverty income ratio, physical activity level and current smoking status in the study population (U_{SG}: 2007 - 2008 (n = 1,838); U_{Osm}: 2009 - 2010 (n = 2,123)) and in a subset of participants free from diabetes mellitus, impaired kidney function, hypertension and diuretic medication (U_{SG}: 2007 - 2008 (n = 852); U_{Osm}: 2009 - 2010 (n = 1,024)). Diabetes flag (diabetes told or antidiabetic meds); metabolic syndrome (any 3 of: elevated waist circumference (> 102 (male), > 88 (female)); elevated fasting plasma glucose (FPG) (≥ 100 mg/dL or antidiabetic medication); elevated triglycerides (≥ 150 or antihyperlipidemic medication); reduced HDL (< 40 (male), < 50 (female) or antihyperlipidemic medication); elevated blood pressure (BP) (systolic ≥ 130 or diastolic ≥ 85 or hypertension medication)); OR: odds ratios; Q: quartile; UCL: upper confidence limit.

* vs Q1
